# Incidence of breast cancer in Chinese women exposed to the 1959–1961 great Chinese famine

**DOI:** 10.1186/s12885-017-3794-3

**Published:** 2017-12-05

**Authors:** Dandan He, Yuan Fang, Marc J. Gunter, Dongli Xu, Yanping Zhao, Jie Zhou, Hong Fang, Wang Hong Xu

**Affiliations:** 1 Center for Disease Control and Prevention of Minhang District, 965 Zhong Yi Road, Shanghai, 201101 China; 2 Department of Epidemiology, School of Public Health, Fudan University; Key Laboratory of Public Health Safety, Ministry of Education (Fudan University), 138 Yi Xue Yuan Road, Shanghai, 200032 China; 3 Section of Nutrition and Metabolism, International Agency for Research on Cancer, 150 Cours Albert Thomas, 69008 Lyon, France

**Keywords:** Breast cancer, Chinese women, Incidence, The great leap forward famine

## Abstract

**Background:**

The association of malnutrition in early life with breast cancer risk has been studied in Europe by investigating survivors of the Dutch Hunger Winter Famine, but not in China. We evaluated the effect of exposure to the 1959–1961 Great Leap Forward famine on subsequent breast cancer risk in Chinese women.

**Methods:**

A total of 59,060 women born in 1955~1966 were recruited from Minhang district, Shanghai, China, during the period 2008 to 2012. A baseline survey was conducted to collect demographic characteristics and known risk factors for breast cancer. Incident breast cancers were identified by conducting record linkage with the Shanghai Cancer Registry up to June 30, 2015, and confirmed through medical records. Cumulative probabilities of cancer incidence were evaluated after adjusting for age, educational level and other confounders. Cox regression models were applied to estimate the hazard ratios (HR) and 95% confidence intervals (CI) of breast cancer.

**Results:**

The overall crude incidence of in situ and invasive breast cancer were 19.2 and 115.0 per 100,000, respectively, in women conceived or born during the famine (1959–1962), slightly higher than those in women born before (1955–1958) (13.2 and 109.8/100,000) and after (1963–1966) (10.4 and 101.5/100,000). Particularly, at age group of 50–52 years when all participants contributed person-year of observations, the age-specific incidence of invasive breast cancer was higher in pre-natal exposed women (123.7/100,000, 95%CI: 94.5–161.9/100,000) than in post-natal exposed (109.6/100,000, 95%CI: 69.1–174.0/100,000) and unexposed women (82.7/100,000, 95%CI: 46.9–145.7/100,000). However, the incidence of cancer in situ was slightly lower in pre-natal exposed women at the age group. Adjusted cumulative probabilities of breast cancer incidence, both in-situ and invasive, were also observed to be higher in women exposed to the famine, however, the difference was not statistically significant.

**Conclusion:**

Our results suggest a possible adverse, but limited, impact of exposure to the Great famine on the risk of breast cancer in Chinese women.

**Electronic supplementary material:**

The online version of this article (10.1186/s12885-017-3794-3) contains supplementary material, which is available to authorized users.

## Background

Breast cancer, the most common malignancy in women worldwide, is responsible for nearly one-fifth of deaths in women aged 40 to 50 years [1]. The incidence of breast cancer has been increasing over the past decades around the world, including in China, a developing country with historically a lower incidence of the malignancy [2]. The upward trend of breast cancer incidence in Chinese women has been attributed to the growing impact of western lifestyles in the country [3]. Over-nutrition in adulthood, particularly when combined with malnutrition in early life, has been suggested to increase the risk of breast cancer in Asian populations [4, 5].

According to Barker’s “fetal origin” hypothesis, adverse intrauterine conditions may have a profound effect on health in later life [6]. Trichopoulos [7] proposed that breast cancer may originate in utero due to exposure to increased concentrations of maternal oestrogens. Both hypotheses were supported by a body of evidence from animal experiments and epidemiological studies. Studies in rodents have shown reductions in tumor occurrence by calorie deprivation [8, 9], which was suggested to modulate the expression of estrogens receptors [10]. However, results derived from human populations are conflicting [11–14]. While several studies observed a lower risk of breast cancer in women with low birth weight, and found that the risk increased with increasing birth weight [11, 12], studies conducted in survivors of the Dutch famine observed an increased risk of breast cancer compared to women unexposed [15–17].

The Dutch famine was a severe but short-term period of malnutrition that occurred in a previously and subsequently well-nourished population [18]. The Great Leap Forward famine in China, on the other hand, was a much more severe event occurring during the period of 1959 to 1961 [19, 20]. Despite the disastrous impact of the famine on Chinese population, its occurrence provides us an opportunity to study the health consequences of malnutrition in early life [21, 22]. However, there is no prior study examining the long-term effect of the Great Famine on the occurrence of breast cancer in Chinese women.

In this study, we compared the incidence of the breast cancer among Chinese women born during and after the Great Famine with those born before the event, and thus evaluated the potential effect of malnutrition in early life on breast cancer risk.

## Methods

### Subjects and study design

This retrospective study used data from a former breast cancer screening program provided to female permanent residents of Shanghai who were living in communities of Minhang district, Shanghai, China, at the time of interview. As described in our previous report [23], 149,577 women, accounting for 65% of a total of 231,069 women at age of 40–74 years old and free of breast cancer in the district, participated in the screening program during the period of 2008 and 2012.

In-person interviews were conducted for all participants using a structured questionnaire to collect information on demographic characteristics, reproductive factors, family history of breast cancer and prior diagnosis of any breast diseases (see Additional file 1). After excluding those born before 1955 and after 1966, a total of 59,060 women born between 1955 and 1966 and free of breast cancer were included in the current study.

This study was approved by the Institutional Review Board (IRB) of the Center of Disease Prevention and Control of Minhang district, Shanghai, China. Verbal consent was obtained from each participant.

### Identification of incident breast cancer

All subjects of the study were followed-up by a record linkage with the Shanghai Cancer Registry and the Shanghai Vital Statistics. The start time of following-up was from the date of recruitment which was from May 23, 2008 to Sep 30, 2012 in calendar time or from 42 to 57 years old with respect to age of the subjects. In April 2016, the record linkage was conducted for all subjects using the unique ID number, a number given to each Chinese citizen by birth and remaining unchanged in whole life, to identify the incident breast cancer and obtain vital status up to June 30, 2015. Information about the Shanghai Cancer Registry system has been described in detail elsewhere [24–26]. Briefly, the Shanghai Cancer Registry was established in 1963, covering 100% permanent residents of urban Shanghai before 2001 and of both urban and rural areas of Shanghai thereafter.

The incident breast cancer cases were identified according to ICD-10 codes of C50. Basic demographic characteristic, tumor site, pathological type and stage of the cancer were available in the system. All incident breast cancer cases identified through linkage were subsequently confirmed by medical record examination.

### Statistical analysis

Since the Great Chinese Famine occurred in 1959 and continued until 1961, exposure to the Famine was defined based on birth year of our subjects. As did previous studies [27], women born between January 1, 1959 and December 31, 1962 were considered to have been pre-natal exposed to the Famine (*n* = 17,772), while those born before 1959 were regarded post-natal exposure (*n* = 25,836) and those born after 1962 were treated as unexposed group (*n* = 15,452). The post-natal exposure group was used as the reference group. Time at risk started at the date of baseline survey and ended at the date of diagnosis of breast cancer, date of death, or ending date of following-up (June 30, 2015), whichever occurred first.

Chi-square tests were used to compare demographic characteristics of study participants across the three birth-year subgroups. The direct adjusted cumulative probabilities of breast cancer incidence were calculated and curved based on a stratified Cox regression model proposed by Zhang et al. [28]. Cox proportional hazard modelling was used to estimate the hazard ratios (HR) and 95% confidence intervals (CIs) of breast cancer related to the exposure to the Famine. Known risk factors of breast cancer such as age (as a continuous variable), educational level (Primary school or below / Middle School / Technical school / High school / College or above, as dummy variables), marital status (married / other status), regular menstrual cycle (yes / no), breastfeeding (ever / never) and family history of breast cancer (ever / never) were adjusted in the models as potential confounding factors for their unbalanced distributions among the three subgroups. A sensitivity analysis was conducted by re-defining the cohort periods as 1955–1959, 1960–1962 and 1963–1966 to minimize the potential misclassification bias since those born in early 1959 were actually conceived in 1958.

All tests were two sided, and *p* values less than 0.05 were considered statistically significant. All statistics were analyzed using SAS statistical package (version 9.3).

## Results

Presented in Table 1 are baseline demographic characteristics and reproductive factors of our subjects. No significant difference was observed among subgroups with respect to age at menarche, infertility, age at first child’s birth and exogenous estrogen use (*p* > 0.05). The post-natal exposure group, however, were more likely to have a lower level of education, later age at menopause, an irregular menstrual cycle and a family history of breast cancer (*p* < 0.0001), but less likely in marriage and breast fed compared to other two groups.

**Table 1:** Comparison of demographic and reproductive factors by birth year in Chinese women

	Birth year			*p value*
	1955–1958 (*N* = 25,836)	1959–1962 (*N* = 17,772)	1963–1966 (*N* = 15,452)	
Participant rates (%)	68.7	61.2	54.6	
Age (Mean ± SD)	52.7 ± 1.9	49.1 ± 1.9	45.2 ± 1.8	<0.0001
Educational level (N, %)				
Primary school or below	5448 (21.1)	2307 (13.0)	1521 (9.8)	
Junior high School	11,926 (46.2)	8669 (48.8)	9466 (61.3)	
Senior high school	637 (2.5)	511 (2.9)	3336 (21.6)	
Technical school	7334 (28.4)	5905 (33.2)	693 (4.5)	
College or above	491 (1.9)	380 (2.1)	436 (2.8)	*<0.0001*
Marriage status (N, %)				
Single	80 (0.3)	52 (0.3)	32 (0.2)	
Married	25,070 (97.0)	17,279 (97.2)	15,052 (97.4)	
Remarried	351 (1.4)	243 (1.4)	226 (1.5)	
Separate/divorced	163 (0.6)	119 (0.7)	111 (0.7)	
Widow	172 (0.7)	79 (0.4)	31 (0.2)	*0.0005*
Age at menarche <12 years (N, %)	66 (0.3)	50 (0.3)	46 (0.3)	*0.41*
Irregular cycle (N, %)	7040 (27.3)	3374 (19.0)	2488 (16.1)	*<0.0001*
Age at menopause ≥55 years (N, %)	96 (0.37)	32 (0.18)	12 (0.08)	*<0.0001*
Infertility (N, %)	446 (1.7)	282 (1.6)	241 (1.6)	*0.17*
Age at first birth >35 years (N, %)	122 (0.5)	90 (0.5)	42 (0.5)	*0.86*
Breastfeeding (N, %)	19,311 (74.7)	14,780 (83.2)	13,241 (85.7)	*<0.0001*
Estrogen use (N, %)	405 (1.6)	219 (1.2)	223 (1.4)	*0.16*
Family history of breast cancer (N, %)	297 (1.2)	136 (0.8)	101 (0.7)	*<0.0001*

During a total of 302, 019 person years of following-up, 373 incident breast cancer patients were identified, including 43 with cancer in situ and 330 with cancer invasive. As shown in Table 2, the incidence of breast cancer in situ and invasive were 19.2 (95%CI: 11.9–30.8) and 115.0 (95%CI: 94.7–139.6) per 100,000, respectively, in women conceived or born during the Great Famine (1959–1962), slightly higher than those in women born before (1955–1958) [13.2 (95%CI: 8.3–20.9) and 109.8 (95%CI: 93.6–128.8) per 100,000] and after the famine (1963–1966) [10.4 (95%CI: 5.2–20.8) and 101.5 (95%CI: 81.3–126.8) per 100,000].

**Table 2:** Incidence rates of breast cancer by birth year in Chinese women

	All subjects (*N* = 59,060)	Birth year		
		1955–1958 (*N* = 25,836)	1959–1962 (*N* = 17,772)	1963–1966 (*N* = 15,452)
Cancer in situ				
Person-years	302,019	136,574	88,645	76,800
No. of cases	43	18	17	8
Incidence (95%CI)	14.2 (10.6, 19.2)	13.2 (8.3, 20.9)	19.2 (11.9, 30.8)	10.4 (5.2, 20.8)
Adjusted HR (95%CI)		1.00	1.06 (0.73, 1.51)	0.95 (0.53, 1.71)
Invasive cancer				
Person-years	302,129	136,620	88,691	76,817
No. of cases	330	150	102	78
Incidence (95%CI)	109.2 (98.0, 121.7)	109.8 (93.6, 128.8)	115.0 (94.7, 139.6)	101.5 (81.3, 126.8)
Adjusted HR (95%CI)		1.00	1.85 (0.69, 1.98)	1.36 (0.25, 7.34)
All breast cancer				
Person-years	302,019	136,574	88,645	76,800
No. of cases	373	168	119	86
Incidence (95%CI)	123.5 (111.6, 136.7)	123.0 (105.7, 143.1)	134.2 (112.2, 160.7)	111.9 (90.6, 138.3)
Adjusted HR (95%CI)		1.00	1.13 (0.80, 1.58)	0.98 (0.57, 1.73)

^a^ Adjusted for age (as a continuous variable), educational level (Primary school or below / Middle School / Technical school / High school / College or above, dummy variables), in marriage (yes /no), regular menstrual cycle (yes / no), breastfeeding (ever / never) and family history of breast cancer (yes / no)

After adjusting for potential confounders, we found that the prenatal exposure group had a slightly elevated hazard ratio (HR) of cancer invasive (HR being 1.85, 95%CI: 0.69–1.98) and cancer in situ (HR being 1.06, 95%CI: 0.73–1.51) compared to the post-natal exposure group, while those unexposed to the Famine had a moderate lower risk of cancer in situ (HR being 0.95, 95%CI: 0.53–1.71), but a slightly higher risk of invasive breast cancer (HR being 1.36, 95%CI: 0.25–7.34). However, the associations did not reach statistical significance (Table 2).

We further calculated crude breast cancer incidence by age groups (< 50, 50–52, and ≥53 years). As shown in Table 3, in age group of 50–52 years during which the three subgroups contributed to person years of observation, the incidence of invasive breast cancer was higher in pre-natal exposed women than in post-natal exposed or unexposed women. However, the incidence of cancer in situ was slightly lower in the post-natal group. In age groups of <50 years or ≥53 years, although the wide age range may lead to residual confounding effect, the incidence of breast cancer was also slightly higher in pre-natal exposed women.

**Table 3:** Age-specific incidence rates of breast cancer by birth year in Chinese women

	< 50 years old			50~52 years old			≥ 53 years old		
	PYs	No. of cases	Incidence (95%CI)	PYs	No. of cases	Incidence (95%CI)	PYs	No. of cases	Incidence (95%CI)
Cancer in situ									
By birth year									
1955–1958	172	0	0.0	16,415	3	18.3 (5.9, 56.6)	119,986	15	12.5 (7.5, 20.7)
1959–1962	17,395	5	28.7 (11.9, 68.9)	42,818	4	9.3 (3.5, 24.8)	28,431	8	28.1 (14.0, 56.0)
1963–1966	62,302	6	9.6 (4.3, 21.4)	14,497	2	13.8 (3.4, 54.9)	–	–	–
Overall	79,870	11	13.8 (7.6, 24.8)	73,731	9	12.2 (6.3, 23.4)	148,418	23	15.5 (10.3, 23.2)
Invasive cancer									
By birth year									
1955–1958	172	0	0	16,418	18	109.6 (69.1, 174.0)	120,030	132	109.9 (92.7, 130.4)
1959–1962	17,399	23	132.2 (87.8, 198.9)	42,838	53	123.7 (94.5, 161.9)	28,453	26	91.3 (62.2, 134.2)
1963–1966	62,314	66	105.9 (83.2, 134.8)	14,503	12	82.7 (46.9, 145.7)	–	–	–
Overall	79,886	89	111.4 (90.5, 137.1)	73,760	83	112.5 (90.7, 139.5)	148,483	158	106.4 (91.0, 124.4)
All breast cancer									
By birth year									
1955–1958	172	0	0	16,416	21	127.9 (83.4, 196.2)	119,987	147	122.5 (104.2, 144.0)
1959–1962	17,395	28	160.9 (111.1, 233.1)	42,818	57	133.1 (102.7, 172.6)	28,431	34	119.6 (85.5, 167.4)
1963–1966	62,302	72	115.6 (91.7, 145.6)	14,497	14	96.6 (57.2, 163.1)	–	–	–
Overall	79,870	100	125.2 (102.9, 152.3)	73,731	92	124.8 (101.7, 153.1)	148,418	181	121.9 (105.4, 141.1)

**Fig. 1:**
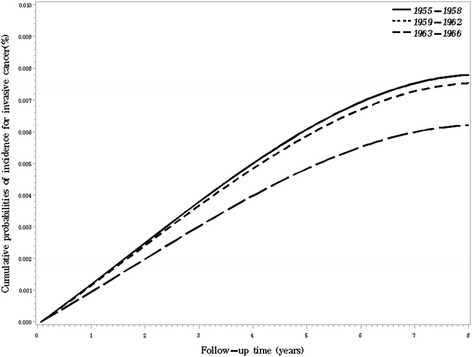
Cumulative probabilities of invasive breast cancer incidence by birth year in Chinese women. Adjusted for age (as a continuous variable), educational level (Primary school or below / Middle School / Technical school / High school / College or above, dummy variables), age at menarche (<12 / ≥12 years old), regular menstrual cycle (yes / no) estrogen use (ever / never) and family history of breast cancer (ever / never)

**Fig. 2:**
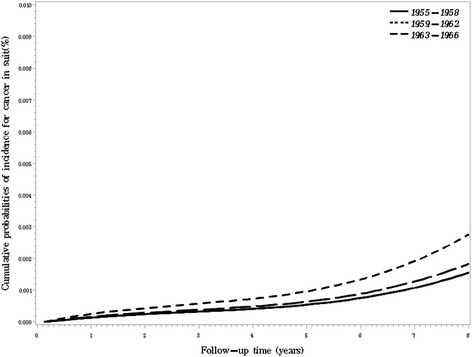
Cumulative probabilities of breast cancer in situ incidence by birth year in Chinese women. Adjusted for age (as a continuous variable), educational level (Primary school or below / Middle School / Technical school / High school / College or above, dummy variables), in marriage (yes / no), regular menstrual cycle (yes / no), breastfeeding (ever / never) and family history of breast cancer (ever / never)

We curved the adjusted cumulative probabilities of breast cancer incidence along with follow-up time by birth years in Fig. 1 and Fig. 2. Women conceived or born during the Great Famine had the highest cumulative probabilities of breast cancer in situ along with the time of following-up, while the post-natal exposure group had the highest cumulative probabilities of invasive breast cancer. We further curved the incidence of breast cancer along with age by the three groups, and found that pre-natal exposure group had higher incidence of invasive cancer before 52 years old and higher incidence of cancer in situ at all ages (figure not shown).

Sensitivity analyses observed similar results. As presented in Table 4, the crude incidence of breast cancer in situ and invasive were 20.5 (95%CI: 11.9–35.3) and 115.2 (95%CI: 91.6–144.9) per 100,000, respectively, in redefined pre-natal exposure group (1960–1962), higher than those in women born before (1955–1959) and after the famine (1963–1966). In age group of 50–52 years old, a higher incidence of breast cancer was also observed in pre-natal exposure women.

**Table 4:** Sensitivity analysis of age-specific incidence rates of breast cancer by birth year in Chinese women

	Incidence (95%CI)	<50 years old			50~52 years old			≥ 53 years old		
		PYs	No. of cases	Incidence (95%CI)	PYs	No. of cases	Incidence (95%CI)	PYs	No. of cases	Incidence (95%CI)
Cancer in situ										
By birth year										
1955–1959	13.6 (8.9, 20.6)	1457	0	0	25,904	3	11.6 (3.7, 35.9)	134,518	19	14.1 (9.0, 22.1)
1960–1962	20.5 (11.9, 35.3)	16,109	5	31.0 (12.9, 74.5)	33,330	4	12.0 (4.5, 31.9)	13,900	4	28.8 (10.8, 76.7)
1963–1966	10.4 (5.2, 20.8)	62,302	6	9.6 (4.3, 21.4)	14,497	2	13.8 (3.4, 55.15)	–	–	–
Overall	14.2 (10.6, 19.2)	79,870	11	13.8 (7.6, 24.9)	73,731	9	12.2 (6.4, 23.4)	148,418	23	15.5 (10.3, 23.3)
Invasive cancer										
By birth year										
1955–1959	110.5 (95.5, 127.9)	1457	1	68.6 (9.7, 487.1)	25,906	29	111.9 (77.8, 161.1)	134,564	149	110.7 (94.3, 130.0)
1960–1962	115.2 (91.6, 144.9)	16,114	22	136.5 (89.9, 207.3)	33,351	42	125.9 (93.1, 170.4)	13,918	9	
1963–1966	101.5 (81.3, 126.8)	62,314	66	105.9 (83.2, 134.8)	14,503	12	82.7 (46.9, 145.7)	–	–	–
Overall	109.2 (98.0, 121.7)	79,885	89	111.4 (90.5, 137.3)	73,759	83	112.5 (90.7, 139.5)	148,483	158	106.4 (91.0, 124.4)
All breast cancer										
By birth year										
1955–1959	124.2 (108.1, 142.6)	1457	1	68.6 (9.7, 487.1)	25,903	32	123.5 (87.4, 174.6)	134,518	168	124.9 (107.4, 145.3)
1960–1962	135.8 (109.9, 167.7)	16,109	27	167.6 (114.9, 244.4)	33,330	46	138.0 (103.4, 184.3)	13,900	13	93.5 (54.3, 161.1)
1963–1966	111.9 (90.6, 138.3)	62,301	72	115.6 (91.7, 145.6)	14,497	14	96.6 (51.8, 163.0)	–	–	–
Overall	123.5 (111.6, 136.7)	79,870	100	125.2 (102.9, 152.3)	73,731	92	124.8 (101.7, 153.1)	148,418	181	121.9 (105.4, 141.1)

## Discussion

The Great Famine in China, which has been viewed as a “natural experiment” in Chinese population, provides us a unique opportunity to evaluate how energy and nutrient deprivation in early life affects the subsequent risk of breast cancer in Chinese women. In this study including 59,060 Chinese women born in 1955–1966, we found that the women exposed to famine during gestation had a slightly higher incidence of breast cancer compared to those born before and after the famine period. The elevated breast cancer incidence in exposed women was observed at age group of 50–52 years, when all participants contributed person-years of observations. However, the associations did not reach statistical significance, suggesting that the effect of extreme malnutrition in early life on subsequent risk of breast cancer may not be profound in this population.

Several biological mechanisms have been proposed to explain the effect of malnutrition in early life on subsequent risk of breast cancer. According to Barker’s “fetal origin” hypothesis, adverse intrauterine conditions may affect human health in later life [6], possibly through “programming”, a process that permanently changes body structures and functions during the maturation of organs and systems. The fetus may be particularly susceptible to environmental challenges due to rapidly proliferating tissue and growth pathways. Energy deficiency in early life along with lack of essential nutrients can alter gene expression, leading to slowing of growth [29, 30] and elevated risk of diseases in adulthood [22, 31, 32], including female breast cancer [13, 14, 17]. Another hypothesis is specifically related to breast cancer. Based on existing empirical data, Trichopoulos [7] proposed that increased concentrations of oestrogens in pregnancy may increase the probability of future occurrence of breast cancer in female offspring. In other words, it is possible that exposure to decreased concentrations of maternal oestrogens due to malnutrition (such as famine) may decrease subsequent risk of breast cancer in daughters. Caloric deprivation has been observed to decrease the size of ovary as well as the secretion of estrogen in animals [33]. Trichopoulos’s hypothesis may help to understand the potential differences in effects of the prenatal and postnatal exposures, because only those prenatal exposed to the famine may have exposed to decreased concentrations of maternal oestrogens.

Our results, although much weaker than those observed in women exposed to the 1944 to 1945 Dutch Famine in early life [13, 17], somewhat support the “fetal origin” hypothesis of breast cancer. Unlike the Dutch Hunger Winter which occurred in a previously and subsequently well-nourished population, the Great Famine in China happened in a population who was historically under-nourished [34]. Compared to the Dutch Hunger Winter, the Great Leap Forward famine lasted much longer (3 years versus 6 months), involved a far broader geographic area (the whole country of China versus some limited areas of the Netherlands), and caused much higher mortality (a mortality of over 3.0% in China versus a mortality of about 1.5% in the Netherlands during the famine) [20, 35, 36]. Even after the famine, a period of insufficient nutrition, but not malnutrition, persisted in China until the 1980s, [34, 37]. It is reported that China experienced a sharp reduction in grain production in 1959. The caloric intake in Chinese people declined drastically below the minimum threshold for basic life support during the famine, let alone fruits and vegetables [38]. During the Dutch Famine, however, the pregnant or lactating women as well as infants were provided extra foods, maintaining a balanced intake of protein, fat and carbohydrate [39]. It is possible that the persistent insufficient nutrition in Chinese population before and even after the Famine may have led to absence of “catch up growth” [40], resulting in a weaker positive association of malnutrition in early life with subsequent risk of breast cancer.

The changed reproductive factors may also contribute to the higher risk of breast cancer in exposed women. It has been suggested that famine exposure in early life may influence women’s reproductive performance in later life [16, 41]. In this study, we also found that the exposures group had a lower level of education, later age at menopause, irregular menstrual cycle, were more likely to have a family history of breast cancer but less likely in marriage and breast fed, which have been associated with breast cancer risk [42, 43]. It is possible that the effect of exposure to famine may partly mediated by the changed reproductive pattern. These mediators were adjusted in the analysis, possibly leading to underestimation of the risk and the swoop between the incidence rate and adjusted HR. Unfortunately, we could not make further evaluations due to lack of detailed information on menstrual and reproductive factors.

The strengths of this study include the relatively large sample size, long-term following-up, and relative homogeneity in reproductive patterns due to the “one child” family plan policy that persisted for more than 30 years from 1978 to 2016 in China.

However, the study has several limitations. First, we used the birth year rather than individual exposure data to define exposed or unexposed groups, which may have led to misclassification bias. Second, the study was not based on a birth cohort, but just included women born between 1955 and 1966. It was estimated that about 15–30 million people starved to death during the Great Famine [44]. Fetuses, infants, children and adults in poor health condition were more likely to die in the famine [45]. Survival bias cannot be excluded, which may have biased the associations between exposure to the Famine and subsequent risk of breast cancer towards null. Furthermore, women in unexposed group were much younger than those in other two subgroups. They did not reach the peak age at diagnosis with breast cancer, which was usually at 55–59 years old in Chinese women [46], much younger than those in western women [47]. Residual confounding effect of age cannot be eliminated. However, the increased age-specific incidence of breast cancer at 50–52 years in prenatal exposure group partly released our concern because at the age group all three subgroups contributed to person years of observation. Finally, we did not collect detailed information on menopausal status, number of live birth, alcohol consumption, cigarette smoking, body mass index (BMI) and some other risk factors of breast cancer, which may represent important confounding effects. Nevertheless, given low rates of alcohol consumption and cigarette smoking in Chinese women, postmenopausal status in majority of our subjects and possible mediation effects of the factors, these factors were not likely as important confounders in this study.

## Conclusions

In summary, our finding of a non-statistically significant higher incidence of breast cancer in Chinese women exposed to the Great Famine suggests a potential moderate effect of malnutrition in early life on subsequent risk of breast cancer. Longer following-up of this cohort of women is warranted to confirm our results.

## Additional file

Additional file 1: Health Questionnaire for Breast Cancer Screening in Minhang District, Shanghai (DOCX 20 kb)

## Abbreviations

BMI: body mass index
CIs: confidence intervals
HR: hazard ratios
IGF: insulin-like Growth Factor
IRB: Institutional Review Board
