# Supplementary material for: Incidence of breast cancer in Chinese women exposed to the 1959–1961 great Chinese famine
Source: BMC Cancer. 2017 Dec 5;17:824. doi: 10.1186/s12885-017-3794-3 (PMC5718143; doi:10.1186/s12885-017-3794-3)
Supplement: Additional file 1: — Health Questionnaire for Breast Cancer Screening in Minhang District, Shanghai (DOCX 20 kb) [file 12885_2017_3794_MOESM1_ESM.docx]

**Health Questionnaire for Breast Cancer Screening in Minhang District, Shanghai**

Please write your answers in the blanks or tick your choice at the right option

| **ID_________________** | |  |
| --- | --- | --- |
| **Section 1: Basic Information** | |  |
| 1. Name：_________________ |  |  |
| 2. Date of birth： / /  day month year |  |  |
| 3. Birth place： |  |  |
| 4. Place registered as a permanent citizen of China：__________________  |  |  |
| 5. Home address：_________________________________________________  6. Telephone number： |  |  |
| 7. What is your highest level of education?  ①Primary school or below ②Junior high school ③Senior high school  ④Occupational or technical school ⑤College or above |  |  |
| 9. Marital status：  ①Single ②Married ③Separate/divorced ④Window ⑤Remarried |  |  |
| 10. Monthly income of your family during past three months (*yuan* RMB)：  ① <1000 ② 1000～ ③ 2000～ ④ 4000～ ⑤ 6000～ ⑥ ≥10000 |  |  |
| 11. Are you working in：  ①Government section ②Healthcare institute ③School ④Other public organization  ⑤State-owned enterprise ⑥foreign-invested or joint venture enterprise  ⑦Private enterprise ⑧Farmers ⑨Unemployed ⑩Others |  |  |
| **Section 2 Information on breast diseases and related factors** | |  |
| 12. Is your age at menarche less than 12 years old? ①Yes ②No |  | |
| 13. Is your age at menopause older than 55 years old? ①Yes ②No |  | |
| 14. Is your age at first delivery older than 35 years old? ①Yes ②No |  | |
| 15. Have you ever breastfed a child? ①Yes ②No ③ No child |  | |
| 16. Do you have regular menstrual cycles? ①Yes ②No |  | |
| 17. Have you ever used any estrogens? ①Yes ②No |  | |
| 18. Have you ever been told by a doctor that you have moderate or severe breast lobular hyperplasia?  ①Yes ②No ③Unknown |  | |
| 19. Have you ever been told by a doctor that you have an intraductal papilloma?  ①Yes ②No ③Unknown |  | |
| 20. Have you ever been diagnosed with breast cancer? ①Yes ②No |  | |
| 21. Have you ever been told by a doctor that you have [dysfunctional](file:///C:\Users\14357\AppData\Local\youdao\dict\Application\7.5.0.0\resultui\dict\?keyword=dysfunctional)[uterine](file:///C:\Users\14357\AppData\Local\youdao\dict\Application\7.5.0.0\resultui\dict\?keyword=uterine)[bleeding](file:///C:\Users\14357\AppData\Local\youdao\dict\Application\7.5.0.0\resultui\dict\?keyword=bleeding)?  ①Yes ②No ③Unknown |  |  |
| 22．Have you ever been diagnosed with ovarian or endometrial cancer? ①Yes ②No |  | |
| 23．Has any of your family members been diagnosed with breast cancer? ①Yes ②No |  | |
| 24. Have you ever had a nodule or lump in your breast? ①Yes ②No |  | |
| 25. Is there any abnormal change on your nipples, itching, redness or scales? ①Yes ②No |  | |
| 26. Is there any abnormal change on your breast skin, dimpling or wrinkles? ①Yes ②No |  | |
| 27. Do you have discharges from your nipples? ①Yes ②No |  | |
| **Thank you for your time and cooperation!**  Signature of interviewer Date of interview |  | |
